# Crystal structure of 2-[4-(4-chloro­phen­yl)-1-(4-meth­oxy­phen­yl)-2-oxoazetidin-3-yl]benzo[*de*]iso­quinoline-1,3-dione dimethyl sulfoxide monosolvate

**DOI:** 10.1107/S2056989015001425

**Published:** 2015-01-28

**Authors:** Ísmail Çelik, Mehmet Akkurt, Aliasghar Jarrahpour, Javad Ameri Rad, Ömer Çelik

**Affiliations:** a Department of Physics, Faculty of Arts and Sciences, Cumhuriyet University, 06532 Sivas, Turkey; b Department of Physics, Faculty of Sciences, Erciyes University, 38039 Kayseri, Turkey; c Department of Chemistry, College of Sciences, Shiraz University, 71454 Shiraz, Iran; d Department of Physics, Faculty of Education, Dicle University, 21280, Diyarbakir, Turkey; e Science and Technology Application and Research Center, Dicle University, 21280, Diyarbakir, Turkey

**Keywords:** crystal structure, β-lactam ring, 1*H*-benzo[*de*]iso­quinoline-1,3(2*H*)-dione group, disorder, azetidin-2-ones

## Abstract

In the title solvated compound, C_28_H_19_N_2_O_4_·C_2_H_6_OS, the central β-lactam ring is almost planar (r.m.s. deviation = 0.002 Å). It makes dihedral angles of 1.92 (11), 83.23 (12) and 74.90 (10)° with the meth­oxy- and chloro­phenyl rings and the ring plane of the 1*H*-benzo[*de*]iso­quinoline-1,3(2*H*)-dione group [maximum deviation = 0.089 (1)], respectively. An intra­molecular C—H⋯O hydrogen bond closes an *S*(6) ring and helps to establish the near coplanarity of the β-lactam and meth­oxy­benzene rings. In the crystal, the components are linked by C—H⋯O hydrogen bonds, C—H⋯π inter­actions and aromatic π–π stacking inter­actions [centroid-to-centroid distances = 3.6166 (10) and 3.7159 (10) Å], resulting in a three-dimensional network, The dimethyl sulfoxide solvent mol­ecule is disordered over two sets of sites in a 0.847 (2):0.153 (2) ratio.

## Related literature   

For general background to β-lactams, see: Alcaide & Almendros (2004 ▸); Alcala *et al.* (2011 ▸); Li *et al.* (2011 ▸); Long & Turos (2002 ▸); MacIntyre *et al.* (2010 ▸); Rogers & Kelly (1999 ▸); Sawa *et al.* (2006 ▸); Southgate (1994 ▸); Zhang & Zhou (2011 ▸); Zhang *et al.* (2011 ▸). For related structures, see: Atioğlu *et al.* (2014 ▸); Butcher *et al.* (2011 ▸); Jarrahpour *et al.* (2012 ▸); Zarei (2013 ▸).
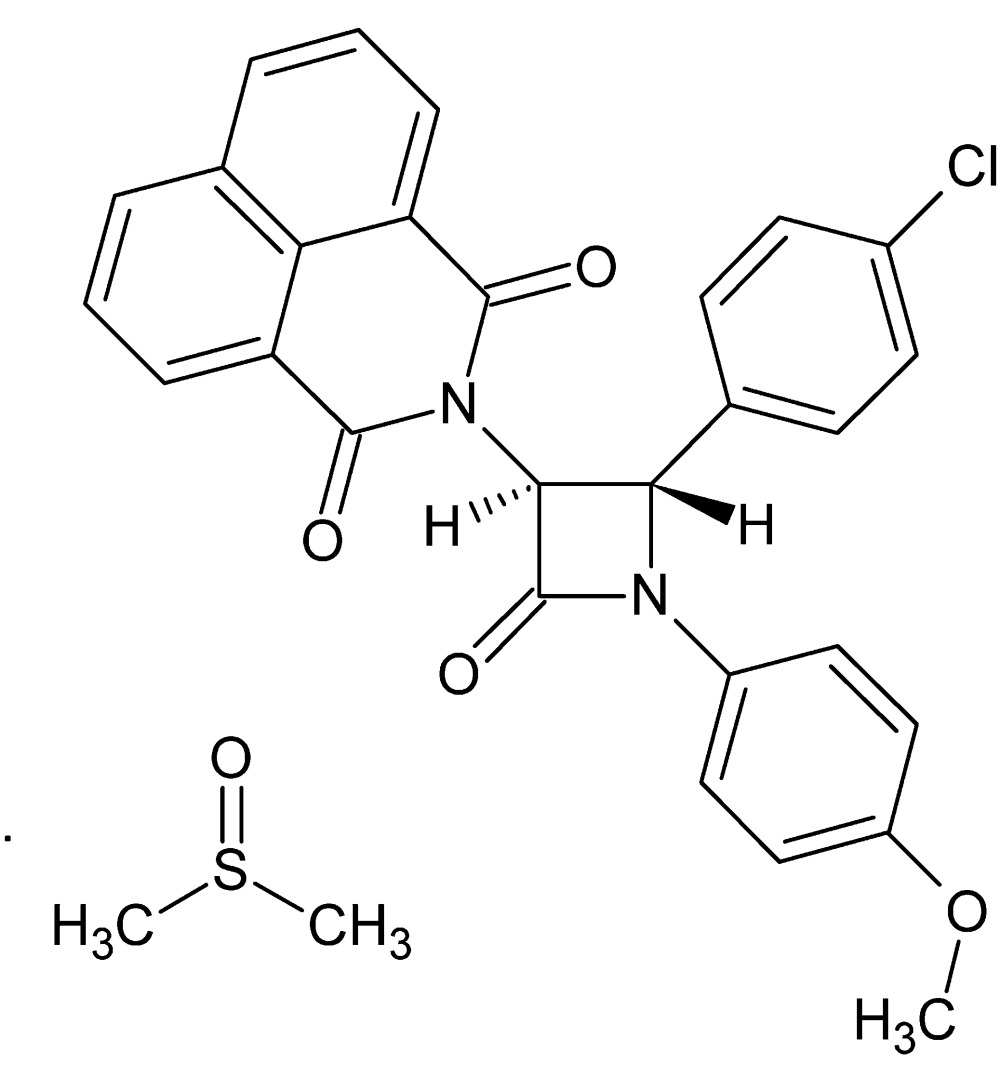


## Experimental   

### Crystal data  

C_28_H_19_ClN_2_O_4_·C_2_H_6_OS
*M* *_r_* = 561.03
Triclinic, 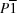

*a* = 7.9925 (3) Å
*b* = 12.1761 (5) Å
*c* = 14.2313 (6) Å
α = 93.549 (2)°
β = 95.520 (2)°
γ = 101.602 (2)°
*V* = 1345.67 (9) Å^3^
*Z* = 2
Mo *K*α radiation
μ = 0.26 mm^−1^
*T* = 296 K
0.45 × 0.30 × 0.20 mm

### Data collection  

Bruker APEXII CCD diffractometer
29975 measured reflections
7737 independent reflections
5777 reflections with *I* > 2σ(*I*)
*R*_int_ = 0.022

### Refinement  

*R*[*F* ^2^ > 2σ(*F* ^2^)] = 0.061
*wR*(*F* ^2^) = 0.191
*S* = 1.05
7737 reflections
359 parameters
6 restraints
H-atom parameters constrained
Δρ_max_ = 0.68 e Å^−3^
Δρ_min_ = −0.52 e Å^−3^

### 

Data collection: *APEX2* (Bruker, 2007 ▸); cell refinement: *SAINT* (Bruker, 2007 ▸); data reduction: *SAINT*; program(s) used to solve structure: *SHELXS2014* (Sheldrick, 2008 ▸); program(s) used to refine structure: *SHELXL2014* (Sheldrick, 2015 ▸); molecular graphics: *ORTEP-3 for Windows* (Farrugia, 2012 ▸); software used to prepare material for publication: *PLATON* (Spek, 2009 ▸).

## Supplementary Material

Crystal structure: contains datablock(s) global, I. DOI: 10.1107/S2056989015001425/hb7354sup1.cif

Structure factors: contains datablock(s) I. DOI: 10.1107/S2056989015001425/hb7354Isup2.hkl

Supporting information file. DOI: 10.1107/S2056989015001425/hb7354Isup3.cml

**Fig. 1.**
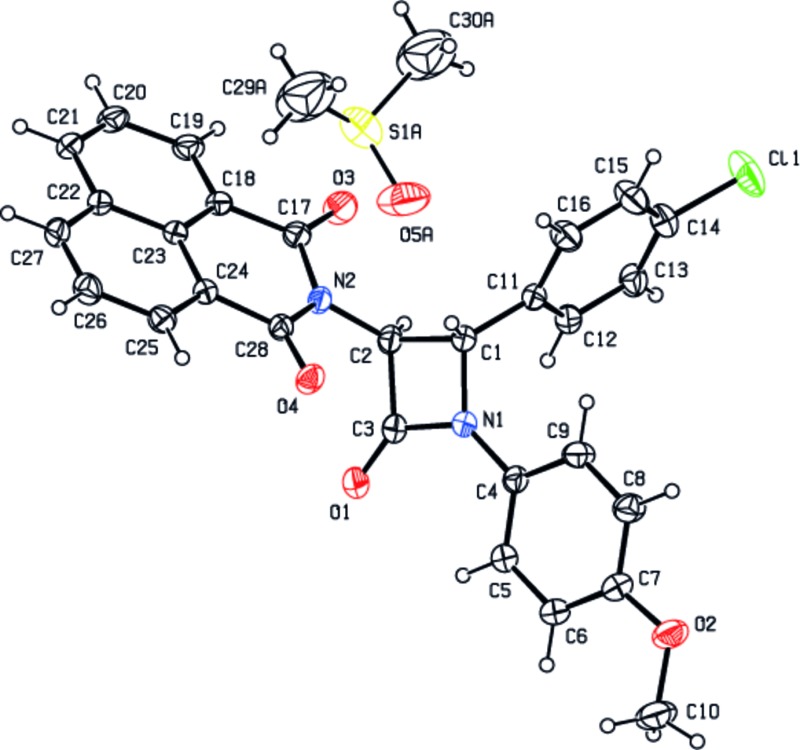
Perspective view of the molecular structure of the title compound with displacement ellipsoids for non-H atoms drawn at the 30% probability level. Only the major component of the disordered solvent molecule is displayed.

**Fig. 2.**
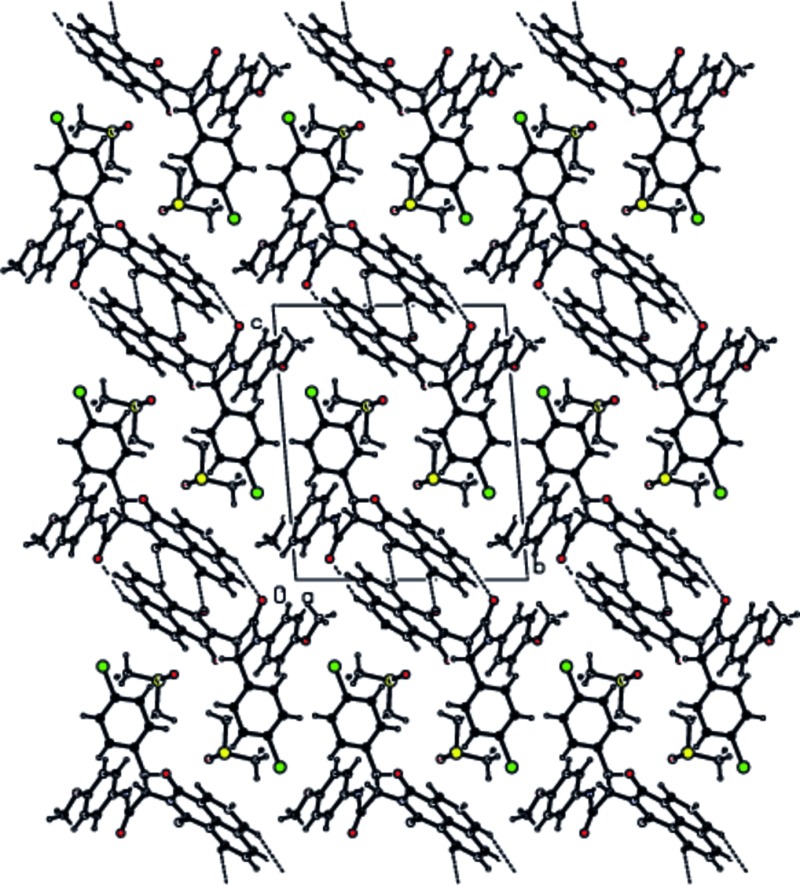
The hydrogen bonding and molecular packing of the title compound along a axis. Only the major component of the disordered solvent molecule is displayed.

**Fig. 3.**
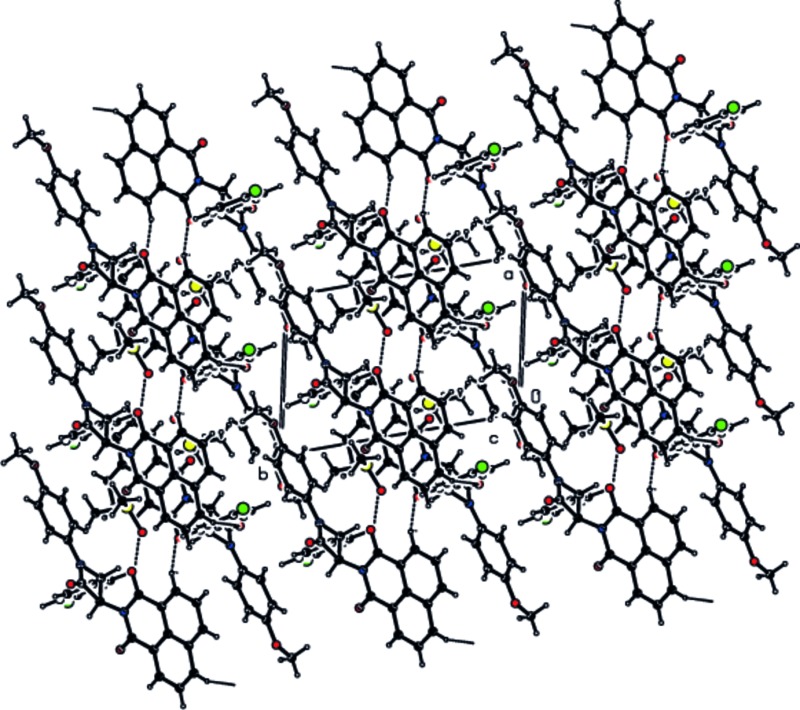
The hydrogen bonding and molecular packing of the title compound along c axis. Only the major component of the disordered solvent molecule is displayed.

. DOI: 10.1107/S2056989015001425/hb7354fig1.tif
Perspective view of the mol­ecular structure of the title compound with displacement ellipsoids for non-H atoms drawn at the 30% probability level. Only the major component of the disordered solvent mol­ecule is displayed.

a . DOI: 10.1107/S2056989015001425/hb7354fig2.tif
The hydrogen bonding and mol­ecular packing of the title compound along *a* axis. Only the major component of the disordered solvent mol­ecule is displayed.

c . DOI: 10.1107/S2056989015001425/hb7354fig3.tif
The hydrogen bonding and mol­ecular packing of the title compound along *c* axis. Only the major component of the disordered solvent mol­ecule is displayed.

CCDC reference: 1044874

Additional supporting information:  crystallographic information; 3D view; checkCIF report

## Figures and Tables

**Table 1:** Hydrogen-bond geometry (, ) *Cg*4 is the centroid of the C11C16 benzene ring.

*D*H*A*	*D*H	H*A*	*D* *A*	*D*H*A*
C5H5O1	0.93	2.57	3.169(3)	122
C21H21O1^i^	0.93	2.52	3.344(2)	148
C25H25O4^ii^	0.93	2.46	3.221(2)	139
C30*A*H30*A* *Cg*4^iii^	0.96	2.88	3.818(10)	167

Symmetry codes: (i) 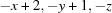; (ii) 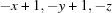; (iii) 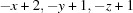.

## supplementary crystallographic information

### S1. Comment

Even more than 70 years after the discovery of penicillin, β-lactam antibiotics remain as one of the most important contributions of science to humanity (Southgate, 1994) and the β-lactam antibiotics have served as a powerful line of defense against bacterial infections (Long, *et al.*, 2002). They have also been used as synthons for the synthesis of various natural and unnatural products (Alcaide & Almendros, 2004). On the other hand, cyclic imides have received special attraction due to their widely potential pharmaceutical applications (Zhang & Zhou, 2011). Isoquinolindione (naphthalimide) derivatives are cyclic imides to be of interest due to their useful photophysical and biological properties that offer promise for medical applications as free radical scavengers (Zhang, *et al.*, 2011), potential photoredox anticancer agents (MacIntyre, *et al.*, 2010), fluorescent labels (Sawa, *et al.*, 2006), photosensitizers (Rogers & Kelly, 1999) and imaging agents (Alcala *et al.*, 2011). Many of these properties are related to 1,8- naphthalimides planar shape and optimal size that makes them efficient DNA intercalating agents with high antitumor activity (Li *et al.*, 2011; Zarei, 2013).

In the title compound (Fig. 1), the β-lactam ring (N1/C1–C3) is nearly planar [r.m.s. deviation = 0.002 Å]. It makes dihedral angles of 1.92 (11), 83.23 (12) and 74.90 (10)° with the methoxy and choloro phenyl rings (C4–C9 and C11–C16) and the ring plane (N2/C17–C28) of the 1*H*-benzo[*de*]isoquinoline-1,3(2*H*)-dione group which is nearly planar [max. deviations = -0.089 (1) Å for N2 and 0.044 (2) Å for C24], respectively.

All bond lengths and bond angles are normal and comparable with those reported for related compounds (Butcher *et al.*, 2011; Atioğlu *et al.*, 2014; Jarrahpour *et al.*, 2012).

Molecular conformation is stabilized by intramolecular C—H···O hydrogen bonds (Table 1). In the crystal, molecules are linked by intermolecular C—H···O hydrogen bonds, forming three dimensional network (Table 1, Figs. 2 & 3).

Furthermore, one weak C—H···π interaction (Table 1) and π-π stacking interactions [*Cg*2···*Cg*6 (2 - *x*,1 - *y*,-*z*) = 3.6166 (10) Å and *Cg*5···*Cg*6(2 - *x*,1 - *y*,-*z*) = 3.7159 (10) Å; where *Cg*2, *Cg*5 and *Cg*6 are centroids of the N2/C17/C18/C23/C24/C28 central pyridine ring and the C18–C23 and C22—C27 benzene rings of the 1*H*-benzo[*de*]isoquinoline-1,3(2*H*)-dione group, respectively] also partially take part in the stabilization of the structure.

### S2. Experimental

4-Chlorophenyl-*N*-(4-methoxyphenyl)methanimine (1 mmol), triethylamine (5 mmol), 2-(1,3-dioxo-1*H*-benzo[*de*]isoquinolin-2(3*H*)-yl)acetic acid (1.50 mmol) and tosyl chloride (1.50 mmol) were added to anhydrous CH_2_Cl_2_ (5 ml) and the mixture was stirred at room temperature for 24 h. The mixture was washed with HCl 1 N (2×20 ml), saturated aqueous NaHCO_3_ solution (50 ml) and brine (20 ml). The organic layer was dried (Na_2_SO_4_) and the solvent was removed to give the product as a white solid·It was then purified by recrystallization from DMSO to afford colourless triclinic crystals (Yield 75%); Mp: 528–530 K; IR (KBr, cm^-1^): 1774 (CO β-lactam),1704 (CO Naph), 1666 (CO Naph); ^1^H-NMR (250 MHz, DMSO-d_6_) δ 1.27 (CH_3_ t, 3H, J = 6.75), 3.95 (CH_2_ q, 2H, J = 6.75), 5.69 (CH β-lactam d, 1H, J = 2.75), 5.94 (CH β-lactam d, 1H, J = 2.75), 6.91 (aromat d, 2H, J = 9.00), 7.19 (aromat d, 2H, J = 9.00), 7.79–7.89 (ArH, m, 4H), 8.24 (aromat d, 2H, J = 9.00), 8.43–8.50 (ArH, m, 4H); ^13^C-NMR (62 MHz, DMSO-d_6_) δ 163.22 (CO β-lactam), 162.13 (CO Naph), 155.04, 147.47, 144.82, 134.88, 131.22, 131.17, 130.50, 128.10, 127.43, 127.30, 123.95, 121.54, 118.29, 115.04, (aromatic carbons), 63.41 (C β-lactam), 63.19 (C β-lactam), 58.08 (CH_2_—O), 14.56 (CH_3_); GC—MS m/*z* = 507 [*M*+].

### S3. Refinement

H atoms were located in calculated positions with C—H = 0.93 - 0.98 Å, and refined using a riding model with *U*_iso_(H) = 1.2 or 1.5*U*_eq_(C). The (0 1 0), (10 - 4 3), (9 - 4 5), (0 0 1), (0 3 5), (0 1 6), (2 2 4), (1 - 5 2), (3 1 5), (-3 3 3), (2 1 6), (-2 4 8), (0 - 3 1), (-2 2 7), (1 - 5 1), (-2 3 4), (3 - 6 9), (2 0 5), (6 3 3), (-2 - 5 2), (-3 5 3), (-3 - 10 7), (-1 0 11) and (-2 - 8 4) reflections were omitted owing to bad disagreement. The crystal quality and data was not good enough. All the atoms of the dimethyl sulfoxide (DMSO) solvent molecule are disordered over two sets of sites in a 0.847 (2):0.153 (2) ratio.

### Crystal data

**Table:**

C_28_H_19_ClN_2_O_4_·C_2_H_6_OS	*Z* = 2
*M**_r_* = 561.03	*F*(000) = 584
Triclinic, *P*1	*D*_x_ = 1.385 Mg m^−^^3^
Hall symbol: -P 1	Mo *K*α radiation, λ = 0.71073 Å
*a* = 7.9925 (3) Å	Cell parameters from 9923 reflections
*b* = 12.1761 (5) Å	θ = 2.9–29.9°
*c* = 14.2313 (6) Å	µ = 0.26 mm^−^^1^
α = 93.549 (2)°	*T* = 296 K
β = 95.520 (2)°	Prism, colourless
γ = 101.602 (2)°	0.45 × 0.30 × 0.20 mm
*V* = 1345.67 (9) Å^3^	

### Data collection

**Table:**

Bruker APEXII CCD diffractometer	5777 reflections with *I* > 2σ(*I*)
Radiation source: sealed tube	*R*_int_ = 0.022
Graphite monochromator	θ_max_ = 30.0°, θ_min_ = 2.2°
φ and ω scans	*h* = −11→11
29975 measured reflections	*k* = −17→17
7737 independent reflections	*l* = −19→19

### Refinement

**Table:**

Refinement on *F*^2^	6 restraints
Least-squares matrix: full	Hydrogen site location: inferred from neighbouring sites
*R*[*F*^2^ > 2σ(*F*^2^)] = 0.061	H-atom parameters constrained
*wR*(*F*^2^) = 0.191	*w* = 1/[σ^2^(*F*_o_^2^) + (0.0977*P*)^2^ + 0.5061*P*] where *P* = (*F*_o_^2^ + 2*F*_c_^2^)/3
*S* = 1.05	(Δ/σ)_max_ < 0.001
7737 reflections	Δρ_max_ = 0.68 e Å^−^^3^
359 parameters	Δρ_min_ = −0.52 e Å^−^^3^

### Special details

**Table:**

Geometry. Bond distances, angles *etc*. have been calculated using the rounded fractional coordinates. All su's are estimated from the variances of the (full) variance-covariance matrix. The cell e.s.d.'s are taken into account in the estimation of distances, angles and torsion angles
Refinement. Refinement on *F*^2^ for ALL reflections except those flagged by the user for potential systematic errors. Weighted *R*-factors *wR* and all goodnesses of fit *S* are based on *F*^2^, conventional *R*-factors *R* are based on *F*, with *F* set to zero for negative *F*^2^. The observed criterion of *F*^2^ > σ(*F*^2^) is used only for calculating -*R*-factor-obs *etc*. and is not relevant to the choice of reflections for refinement. *R*-factors based on *F*^2^ are statistically about twice as large as those based on *F*, and *R*-factors based on ALL data will be even larger.

### Fractional atomic coordinates and isotropic or equivalent isotropic displacement parameters (Å^2^)

**Table:**

	*x*	*y*	*z*	*U*_iso_*/*U*_eq_	Occ. (<1)
Cl1	0.69679 (12)	0.13588 (7)	0.68363 (4)	0.0886 (3)	
S1A	0.83996 (15)	0.62942 (10)	0.36822 (7)	0.0884 (4)	0.847 (2)
S1B	0.7471 (8)	0.6453 (5)	0.4182 (4)	0.0884 (4)	0.153 (2)
O1	0.5935 (2)	0.15324 (13)	0.07604 (10)	0.0572 (5)	
O2	−0.20212 (18)	−0.02113 (14)	0.22243 (12)	0.0620 (5)	
O3	1.06205 (19)	0.37028 (13)	0.29270 (12)	0.0603 (5)	
O4	0.58077 (15)	0.40773 (12)	0.11511 (10)	0.0467 (4)	
N1	0.48129 (19)	0.18953 (13)	0.21909 (10)	0.0411 (4)	
N2	0.82660 (17)	0.38087 (12)	0.19376 (10)	0.0355 (4)	
C1	0.5863 (2)	0.27720 (14)	0.28906 (12)	0.0368 (5)	
C2	0.7342 (2)	0.27719 (14)	0.22466 (12)	0.0381 (5)	
C3	0.5985 (2)	0.19748 (15)	0.15453 (13)	0.0423 (5)	
C4	0.3093 (2)	0.13255 (14)	0.21725 (12)	0.0372 (5)	
C5	0.2294 (3)	0.05633 (17)	0.14244 (13)	0.0469 (6)	
O5A	0.6596 (5)	0.5604 (2)	0.3457 (2)	0.1081 (11)	0.847 (2)
C6	0.0575 (3)	0.00331 (17)	0.14164 (14)	0.0497 (6)	
C7	−0.0327 (2)	0.02482 (16)	0.21594 (14)	0.0450 (5)	
C8	0.0494 (2)	0.09910 (18)	0.29171 (15)	0.0488 (6)	
C9	0.2184 (2)	0.15335 (16)	0.29214 (14)	0.0450 (6)	
C10	−0.2998 (3)	−0.0868 (2)	0.1422 (2)	0.0750 (9)	
C11	0.6159 (2)	0.24211 (14)	0.38776 (12)	0.0365 (4)	
C12	0.6490 (2)	0.13637 (15)	0.40284 (13)	0.0413 (5)	
C13	0.6763 (3)	0.10452 (17)	0.49434 (14)	0.0477 (6)	
C14	0.6676 (3)	0.17782 (19)	0.56949 (14)	0.0524 (6)	
C15	0.6345 (4)	0.2827 (2)	0.55697 (15)	0.0612 (8)	
C16	0.6094 (3)	0.31440 (17)	0.46505 (14)	0.0500 (6)	
C17	1.0008 (2)	0.41714 (14)	0.22957 (12)	0.0376 (5)	
C18	1.1000 (2)	0.51167 (13)	0.18560 (11)	0.0337 (4)	
C19	1.2760 (2)	0.54014 (16)	0.20663 (13)	0.0428 (5)	
C20	1.3723 (2)	0.62737 (18)	0.16236 (15)	0.0495 (6)	
C21	1.2935 (2)	0.68586 (17)	0.09901 (13)	0.0464 (5)	
C22	1.1127 (2)	0.66006 (14)	0.07639 (12)	0.0372 (5)	
C23	1.01511 (19)	0.56994 (13)	0.11954 (10)	0.0316 (4)	
C24	0.83533 (19)	0.53926 (13)	0.09505 (11)	0.0325 (4)	
C25	0.7550 (2)	0.59720 (15)	0.03094 (12)	0.0393 (5)	
C26	0.8512 (3)	0.68702 (16)	−0.01091 (13)	0.0462 (6)	
C27	1.0259 (3)	0.71740 (15)	0.01086 (13)	0.0438 (5)	
C28	0.7350 (2)	0.44063 (14)	0.13389 (11)	0.0341 (4)	
O5B	0.688 (3)	0.6029 (15)	0.3255 (13)	0.1081 (11)	0.153 (2)
C29A	0.8342 (10)	0.7606 (7)	0.3416 (6)	0.183 (3)	0.847 (2)
C30A	0.8821 (12)	0.6486 (7)	0.4874 (7)	0.183 (3)	0.847 (2)
C29B	0.821 (6)	0.786 (2)	0.413 (4)	0.183 (3)	0.153 (2)
C30B	0.904 (5)	0.593 (4)	0.479 (5)	0.183 (3)	0.153 (2)
H1	0.54490	0.34770	0.28880	0.0440*	
H2	0.81630	0.23530	0.25290	0.0460*	
H5	0.29030	0.04040	0.09260	0.0560*	
H6	0.00350	−0.04690	0.09060	0.0600*	
H8	−0.01000	0.11250	0.34290	0.0580*	
H9	0.27160	0.20410	0.34290	0.0540*	
H10A	−0.41530	−0.11360	0.15630	0.1130*	
H10B	−0.30120	−0.04140	0.08940	0.1130*	
H10C	−0.24890	−0.14960	0.12670	0.1130*	
H12	0.65280	0.08650	0.35130	0.0500*	
H13	0.70030	0.03420	0.50420	0.0570*	
H15	0.62890	0.33170	0.60890	0.0730*	
H16	0.58800	0.38550	0.45580	0.0600*	
H19	1.33080	0.50150	0.25020	0.0510*	
H20	1.49120	0.64560	0.17630	0.0590*	
H21	1.35960	0.74340	0.07030	0.0560*	
H25	0.63660	0.57680	0.01530	0.0470*	
H26	0.79580	0.72620	−0.05380	0.0550*	
H27	1.08800	0.77660	−0.01790	0.0530*	
H29A	0.94730	0.80710	0.35510	0.2740*	0.847 (2)
H29B	0.79540	0.76000	0.27560	0.2740*	0.847 (2)
H29C	0.75680	0.78990	0.37900	0.2740*	0.847 (2)
H30A	0.99640	0.69240	0.50420	0.2740*	0.847 (2)
H30B	0.80100	0.68750	0.51270	0.2740*	0.847 (2)
H30C	0.87290	0.57690	0.51320	0.2740*	0.847 (2)
H29D	0.86430	0.82040	0.47590	0.2740*	0.153 (2)
H29E	0.91190	0.79800	0.37310	0.2740*	0.153 (2)
H29F	0.72910	0.81960	0.38830	0.2740*	0.153 (2)
H30D	0.93010	0.63100	0.54170	0.2740*	0.153 (2)
H30E	0.86390	0.51410	0.48360	0.2740*	0.153 (2)
H30F	1.00470	0.60530	0.44680	0.2740*	0.153 (2)

### Atomic displacement parameters (Å^2^)

**Table:**

	*U*^11^	*U*^22^	*U*^33^	*U*^12^	*U*^13^	*U*^23^
Cl1	0.1404 (7)	0.0971 (5)	0.0389 (3)	0.0438 (5)	0.0117 (3)	0.0232 (3)
S1A	0.0995 (7)	0.1062 (7)	0.0621 (5)	0.0320 (6)	0.0056 (4)	−0.0015 (5)
S1B	0.0995 (7)	0.1062 (7)	0.0621 (5)	0.0320 (6)	0.0056 (4)	−0.0015 (5)
O1	0.0713 (10)	0.0540 (8)	0.0402 (7)	−0.0060 (7)	0.0177 (7)	0.0018 (6)
O2	0.0407 (7)	0.0676 (10)	0.0676 (10)	−0.0074 (7)	0.0065 (7)	−0.0120 (8)
O3	0.0527 (8)	0.0609 (9)	0.0618 (9)	0.0030 (7)	−0.0159 (7)	0.0261 (7)
O4	0.0309 (6)	0.0547 (8)	0.0505 (7)	−0.0006 (5)	−0.0019 (5)	0.0152 (6)
N1	0.0403 (7)	0.0450 (8)	0.0327 (7)	−0.0041 (6)	0.0042 (5)	0.0040 (6)
N2	0.0317 (6)	0.0381 (7)	0.0354 (7)	0.0019 (5)	0.0032 (5)	0.0111 (5)
C1	0.0355 (8)	0.0394 (8)	0.0328 (8)	0.0009 (6)	0.0036 (6)	0.0058 (6)
C2	0.0381 (8)	0.0385 (8)	0.0369 (8)	0.0030 (6)	0.0065 (6)	0.0104 (6)
C3	0.0478 (9)	0.0399 (8)	0.0368 (9)	0.0002 (7)	0.0085 (7)	0.0083 (7)
C4	0.0378 (8)	0.0368 (8)	0.0353 (8)	0.0027 (6)	0.0020 (6)	0.0091 (6)
C5	0.0503 (10)	0.0490 (10)	0.0361 (9)	−0.0030 (8)	0.0077 (7)	0.0025 (7)
O5A	0.128 (2)	0.0609 (17)	0.112 (2)	−0.0059 (18)	−0.0486 (19)	0.0060 (15)
C6	0.0515 (10)	0.0459 (10)	0.0431 (10)	−0.0064 (8)	0.0025 (8)	−0.0035 (8)
C7	0.0393 (9)	0.0410 (9)	0.0508 (10)	0.0012 (7)	0.0023 (7)	0.0009 (7)
C8	0.0377 (9)	0.0554 (11)	0.0507 (11)	0.0063 (8)	0.0067 (7)	−0.0065 (8)
C9	0.0383 (9)	0.0486 (10)	0.0440 (10)	0.0051 (7)	−0.0001 (7)	−0.0077 (8)
C10	0.0529 (13)	0.0739 (16)	0.0820 (18)	−0.0143 (11)	0.0018 (12)	−0.0205 (13)
C11	0.0332 (7)	0.0404 (8)	0.0335 (8)	0.0008 (6)	0.0045 (6)	0.0061 (6)
C12	0.0458 (9)	0.0401 (8)	0.0376 (9)	0.0059 (7)	0.0084 (7)	0.0044 (7)
C13	0.0555 (11)	0.0449 (9)	0.0448 (10)	0.0109 (8)	0.0092 (8)	0.0122 (8)
C14	0.0626 (12)	0.0604 (12)	0.0352 (9)	0.0123 (9)	0.0059 (8)	0.0120 (8)
C15	0.0901 (17)	0.0599 (13)	0.0355 (10)	0.0227 (12)	0.0047 (10)	−0.0008 (9)
C16	0.0686 (13)	0.0423 (9)	0.0395 (10)	0.0142 (9)	0.0025 (8)	0.0031 (7)
C17	0.0347 (8)	0.0404 (8)	0.0358 (8)	0.0052 (6)	−0.0021 (6)	0.0056 (6)
C18	0.0310 (7)	0.0361 (7)	0.0313 (7)	0.0025 (6)	0.0011 (5)	−0.0008 (6)
C19	0.0334 (8)	0.0491 (9)	0.0418 (9)	0.0044 (7)	−0.0029 (6)	−0.0031 (7)
C20	0.0315 (8)	0.0594 (11)	0.0495 (10)	−0.0059 (7)	0.0027 (7)	−0.0061 (8)
C21	0.0417 (9)	0.0475 (9)	0.0417 (9)	−0.0108 (7)	0.0095 (7)	−0.0029 (7)
C22	0.0416 (8)	0.0344 (8)	0.0316 (8)	−0.0019 (6)	0.0076 (6)	−0.0016 (6)
C23	0.0324 (7)	0.0323 (7)	0.0276 (7)	0.0017 (5)	0.0037 (5)	−0.0009 (5)
C24	0.0326 (7)	0.0341 (7)	0.0296 (7)	0.0037 (6)	0.0029 (5)	0.0039 (6)
C25	0.0399 (8)	0.0411 (8)	0.0361 (8)	0.0083 (7)	−0.0003 (6)	0.0057 (7)
C26	0.0590 (11)	0.0409 (9)	0.0401 (9)	0.0130 (8)	0.0031 (8)	0.0106 (7)
C27	0.0561 (10)	0.0345 (8)	0.0382 (9)	−0.0002 (7)	0.0105 (7)	0.0060 (7)
C28	0.0310 (7)	0.0389 (8)	0.0312 (7)	0.0040 (6)	0.0020 (5)	0.0069 (6)
O5B	0.128 (2)	0.0609 (17)	0.112 (2)	−0.0059 (18)	−0.0486 (19)	0.0060 (15)
C29A	0.140 (4)	0.184 (6)	0.187 (5)	−0.034 (4)	−0.060 (4)	0.058 (5)
C30A	0.140 (4)	0.184 (6)	0.187 (5)	−0.034 (4)	−0.060 (4)	0.058 (5)
C29B	0.140 (4)	0.184 (6)	0.187 (5)	−0.034 (4)	−0.060 (4)	0.058 (5)
C30B	0.140 (4)	0.184 (6)	0.187 (5)	−0.034 (4)	−0.060 (4)	0.058 (5)

### Geometric parameters (Å, º)

**Table:**

Cl1—C14	1.741 (2)	C22—C23	1.422 (2)
S1A—C30A	1.689 (10)	C22—C27	1.411 (3)
S1A—O5A	1.509 (4)	C23—C24	1.413 (2)
S1A—C29A	1.673 (8)	C24—C25	1.375 (2)
S1B—O5B	1.388 (19)	C24—C28	1.473 (2)
S1B—C30B	1.71 (5)	C25—C26	1.405 (3)
S1B—C29B	1.71 (3)	C26—C27	1.370 (3)
O1—C3	1.203 (2)	C1—H1	0.9800
O2—C10	1.420 (3)	C2—H2	0.9800
O2—C7	1.371 (2)	C5—H5	0.9300
O3—C17	1.210 (2)	C6—H6	0.9300
O4—C28	1.215 (2)	C8—H8	0.9300
N1—C3	1.368 (2)	C9—H9	0.9300
N1—C1	1.477 (2)	C10—H10C	0.9600
N1—C4	1.407 (2)	C10—H10B	0.9600
N2—C28	1.401 (2)	C10—H10A	0.9600
N2—C2	1.445 (2)	C12—H12	0.9300
N2—C17	1.408 (2)	C13—H13	0.9300
C1—C2	1.564 (2)	C15—H15	0.9300
C1—C11	1.504 (2)	C16—H16	0.9300
C2—C3	1.537 (2)	C19—H19	0.9300
C4—C5	1.382 (3)	C20—H20	0.9300
C4—C9	1.385 (2)	C21—H21	0.9300
C5—C6	1.394 (3)	C25—H25	0.9300
C6—C7	1.377 (3)	C26—H26	0.9300
C7—C8	1.384 (3)	C27—H27	0.9300
C8—C9	1.378 (2)	C29A—H29C	0.9600
C11—C12	1.391 (2)	C29A—H29A	0.9600
C11—C16	1.377 (3)	C29A—H29B	0.9600
C12—C13	1.391 (3)	C30A—H30A	0.9600
C13—C14	1.366 (3)	C30A—H30B	0.9600
C14—C15	1.374 (3)	C30A—H30C	0.9600
C15—C16	1.394 (3)	C29B—H29D	0.9700
C17—C18	1.471 (2)	C29B—H29E	0.9600
C18—C23	1.415 (2)	C29B—H29F	0.9600
C18—C19	1.378 (2)	C30B—H30D	0.9600
C19—C20	1.402 (3)	C30B—H30E	0.9600
C20—C21	1.367 (3)	C30B—H30F	0.9500
C21—C22	1.416 (2)		
			
O5A—S1A—C30A	107.4 (3)	O4—C28—N2	119.23 (15)
C29A—S1A—C30A	100.9 (4)	N1—C1—H1	112.00
O5A—S1A—C29A	107.1 (3)	C11—C1—H1	112.00
C29B—S1B—C30B	108 (2)	C2—C1—H1	112.00
O5B—S1B—C29B	106 (2)	C3—C2—H2	110.00
O5B—S1B—C30B	119 (2)	N2—C2—H2	109.00
C7—O2—C10	118.08 (18)	C1—C2—H2	109.00
C3—N1—C4	133.80 (15)	C6—C5—H5	120.00
C1—N1—C4	129.79 (14)	C4—C5—H5	120.00
C1—N1—C3	95.78 (14)	C7—C6—H6	120.00
C2—N2—C17	117.44 (14)	C5—C6—H6	120.00
C17—N2—C28	124.63 (14)	C7—C8—H8	120.00
C2—N2—C28	117.87 (14)	C9—C8—H8	120.00
N1—C1—C11	115.59 (14)	C4—C9—H9	120.00
N1—C1—C2	86.20 (12)	C8—C9—H9	120.00
C2—C1—C11	117.03 (14)	O2—C10—H10C	109.00
C1—C2—C3	85.86 (12)	O2—C10—H10B	109.00
N2—C2—C1	120.95 (14)	H10A—C10—H10C	110.00
N2—C2—C3	119.42 (14)	O2—C10—H10A	109.00
O1—C3—N1	132.51 (17)	H10A—C10—H10B	109.00
N1—C3—C2	91.20 (14)	H10B—C10—H10C	109.00
O1—C3—C2	136.20 (17)	C11—C12—H12	120.00
N1—C4—C9	119.16 (15)	C13—C12—H12	120.00
C5—C4—C9	119.41 (17)	C14—C13—H13	120.00
N1—C4—C5	121.44 (16)	C12—C13—H13	120.00
C4—C5—C6	120.03 (19)	C14—C15—H15	121.00
C5—C6—C7	120.34 (18)	C16—C15—H15	121.00
O2—C7—C8	115.19 (17)	C11—C16—H16	120.00
C6—C7—C8	119.29 (17)	C15—C16—H16	119.00
O2—C7—C6	125.52 (18)	C20—C19—H19	120.00
C7—C8—C9	120.65 (18)	C18—C19—H19	120.00
C4—C9—C8	120.25 (18)	C21—C20—H20	120.00
C12—C11—C16	118.78 (17)	C19—C20—H20	120.00
C1—C11—C16	120.29 (16)	C22—C21—H21	120.00
C1—C11—C12	120.93 (15)	C20—C21—H21	120.00
C11—C12—C13	120.60 (17)	C26—C25—H25	120.00
C12—C13—C14	119.18 (19)	C24—C25—H25	120.00
C13—C14—C15	121.63 (19)	C27—C26—H26	120.00
Cl1—C14—C13	118.83 (17)	C25—C26—H26	120.00
Cl1—C14—C15	119.53 (16)	C22—C27—H27	120.00
C14—C15—C16	118.8 (2)	C26—C27—H27	120.00
C11—C16—C15	121.02 (19)	S1A—C29A—H29B	109.00
O3—C17—C18	123.36 (16)	H29A—C29A—H29C	109.00
O3—C17—N2	120.05 (16)	S1A—C29A—H29C	109.00
N2—C17—C18	116.58 (14)	H29A—C29A—H29B	110.00
C17—C18—C19	119.58 (15)	S1A—C29A—H29A	109.00
C17—C18—C23	119.88 (14)	H29B—C29A—H29C	110.00
C19—C18—C23	120.51 (15)	S1A—C30A—H30C	110.00
C18—C19—C20	119.98 (16)	H30A—C30A—H30C	109.00
C19—C20—C21	120.76 (16)	H30B—C30A—H30C	109.00
C20—C21—C22	120.96 (17)	H30A—C30A—H30B	109.00
C21—C22—C27	122.97 (17)	S1A—C30A—H30A	110.00
C23—C22—C27	118.61 (16)	S1A—C30A—H30B	109.00
C21—C22—C23	118.39 (15)	S1B—C29B—H29D	109.00
C22—C23—C24	119.53 (14)	S1B—C29B—H29E	110.00
C18—C23—C24	121.09 (14)	S1B—C29B—H29F	110.00
C18—C23—C22	119.37 (14)	H29D—C29B—H29E	109.00
C23—C24—C25	120.25 (15)	H29D—C29B—H29F	109.00
C25—C24—C28	119.77 (14)	H29E—C29B—H29F	110.00
C23—C24—C28	119.88 (14)	S1B—C30B—H30D	109.00
C24—C25—C26	120.21 (16)	S1B—C30B—H30E	109.00
C25—C26—C27	120.58 (18)	S1B—C30B—H30F	110.00
C22—C27—C26	120.81 (17)	H30D—C30B—H30E	109.00
N2—C28—C24	116.85 (14)	H30D—C30B—H30F	110.00
O4—C28—C24	123.89 (15)	H30E—C30B—H30F	110.00
			
C10—O2—C7—C6	−7.4 (3)	C6—C7—C8—C9	1.6 (3)
C10—O2—C7—C8	172.23 (19)	O2—C7—C8—C9	−178.04 (18)
C3—N1—C1—C2	−7.68 (13)	C7—C8—C9—C4	−1.2 (3)
C4—N1—C1—C11	62.4 (2)	C1—C11—C12—C13	180.00 (18)
C4—N1—C1—C2	−179.39 (17)	C16—C11—C12—C13	−0.5 (3)
C3—N1—C1—C11	−125.92 (15)	C12—C11—C16—C15	−0.3 (3)
C4—N1—C3—O1	−4.1 (4)	C1—C11—C16—C15	179.2 (2)
C1—N1—C3—C2	7.80 (14)	C11—C12—C13—C14	1.0 (3)
C4—N1—C3—C2	178.97 (19)	C12—C13—C14—C15	−0.8 (4)
C1—N1—C4—C5	176.58 (17)	C12—C13—C14—Cl1	178.46 (17)
C3—N1—C4—C5	8.0 (3)	Cl1—C14—C15—C16	−179.2 (2)
C1—N1—C4—C9	−3.5 (3)	C13—C14—C15—C16	0.0 (4)
C3—N1—C4—C9	−172.02 (19)	C14—C15—C16—C11	0.6 (4)
C1—N1—C3—O1	−175.3 (2)	O3—C17—C18—C19	9.3 (3)
C17—N2—C2—C3	−143.50 (15)	N2—C17—C18—C23	7.7 (2)
C28—N2—C2—C3	39.3 (2)	O3—C17—C18—C23	−173.05 (17)
C28—N2—C17—C18	−13.0 (2)	N2—C17—C18—C19	−169.99 (15)
C28—N2—C2—C1	−64.7 (2)	C17—C18—C23—C22	−179.03 (15)
C2—N2—C28—O4	4.7 (2)	C19—C18—C23—C24	177.96 (15)
C2—N2—C17—O3	−9.2 (2)	C17—C18—C23—C24	0.3 (2)
C17—N2—C28—O4	−172.28 (16)	C19—C18—C23—C22	−1.4 (2)
C28—N2—C17—O3	167.75 (17)	C23—C18—C19—C20	−0.1 (3)
C2—N2—C17—C18	170.06 (14)	C17—C18—C19—C20	177.60 (17)
C2—N2—C28—C24	−173.56 (14)	C18—C19—C20—C21	0.8 (3)
C17—N2—C2—C1	112.52 (17)	C19—C20—C21—C22	0.0 (3)
C17—N2—C28—C24	9.5 (2)	C20—C21—C22—C23	−1.5 (3)
N1—C1—C11—C16	−139.21 (18)	C20—C21—C22—C27	−179.41 (18)
N1—C1—C11—C12	40.3 (2)	C21—C22—C23—C24	−177.24 (15)
C2—C1—C11—C16	121.47 (19)	C27—C22—C23—C18	−179.85 (15)
C11—C1—C2—C3	123.69 (15)	C21—C22—C23—C18	2.1 (2)
N1—C1—C2—C3	6.81 (12)	C23—C22—C27—C26	0.0 (3)
C11—C1—C2—N2	−114.25 (17)	C27—C22—C23—C24	0.8 (2)
C2—C1—C11—C12	−59.0 (2)	C21—C22—C27—C26	177.94 (18)
N1—C1—C2—N2	128.87 (15)	C18—C23—C24—C28	−3.9 (2)
C1—C2—C3—O1	175.9 (2)	C22—C23—C24—C25	−0.9 (2)
N2—C2—C3—N1	−130.79 (15)	C22—C23—C24—C28	175.38 (14)
N2—C2—C3—O1	52.5 (3)	C18—C23—C24—C25	179.75 (15)
C1—C2—C3—N1	−7.35 (13)	C23—C24—C25—C26	0.2 (2)
C5—C4—C9—C8	−0.5 (3)	C25—C24—C28—N2	175.73 (15)
N1—C4—C9—C8	179.60 (17)	C23—C24—C28—O4	−178.75 (16)
C9—C4—C5—C6	1.6 (3)	C23—C24—C28—N2	−0.6 (2)
N1—C4—C5—C6	−178.42 (18)	C28—C24—C25—C26	−176.07 (16)
C4—C5—C6—C7	−1.2 (3)	C25—C24—C28—O4	−2.4 (3)
C5—C6—C7—O2	179.20 (19)	C24—C25—C26—C27	0.6 (3)
C5—C6—C7—C8	−0.4 (3)	C25—C26—C27—C22	−0.7 (3)

### Hydrogen-bond geometry (Å, º)

Cg4 is the centroid of the C11–C16 benzene ring.

**Table:**

*D*—H···*A*	*D*—H	H···*A*	*D*···*A*	*D*—H···*A*
C5—H5···O1	0.93	2.57	3.169 (3)	122
C21—H21···O1^i^	0.93	2.52	3.344 (2)	148
C25—H25···O4^ii^	0.93	2.46	3.221 (2)	139
C30*A*—H30*A*···*Cg*4^iii^	0.96	2.88	3.818 (10)	167

Symmetry codes: (i) −*x*+2, −*y*+1, −*z*; (ii) −*x*+1, −*y*+1, −*z*; (iii) −*x*+2, −*y*+1, −*z*+1.
