# Supplementary figures and images for: Crystal structure of 2-[4-(4-chloro­phen­yl)-1-(4-meth­oxy­phen­yl)-2-oxoazetidin-3-yl]benzo[de]iso­quinoline-1,3-dione dimethyl sulfoxide monosolvate
Source: Acta Crystallogr E Crystallogr Commun. 2015 Jan 28;71(Pt 2):o129–30. doi: 10.1107/S2056989015001425 (PMC4384601; doi:10.1107/S2056989015001425)

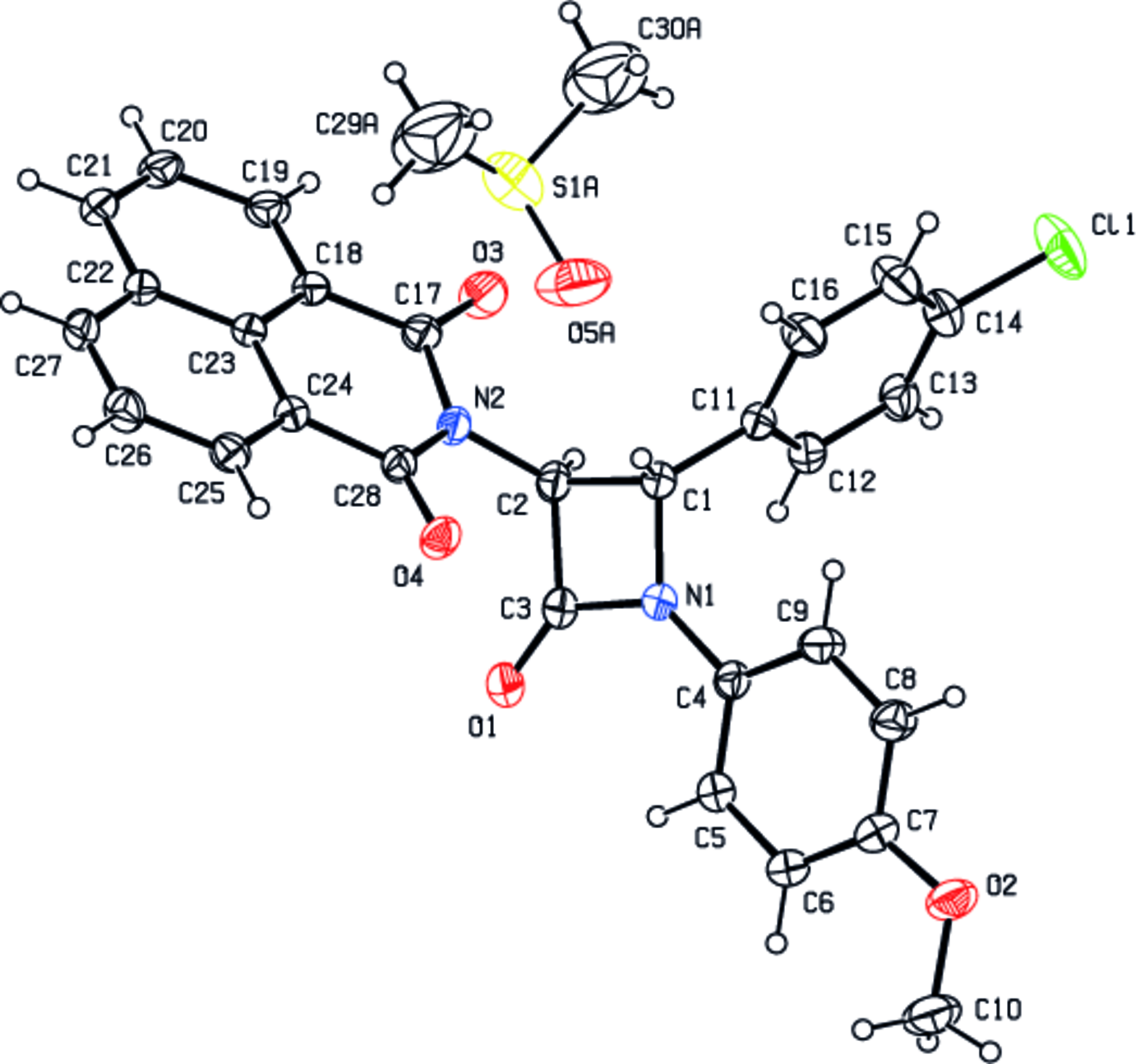

Supplement: Supplementary file 4 [file e-71-0o129-fig1.tif]

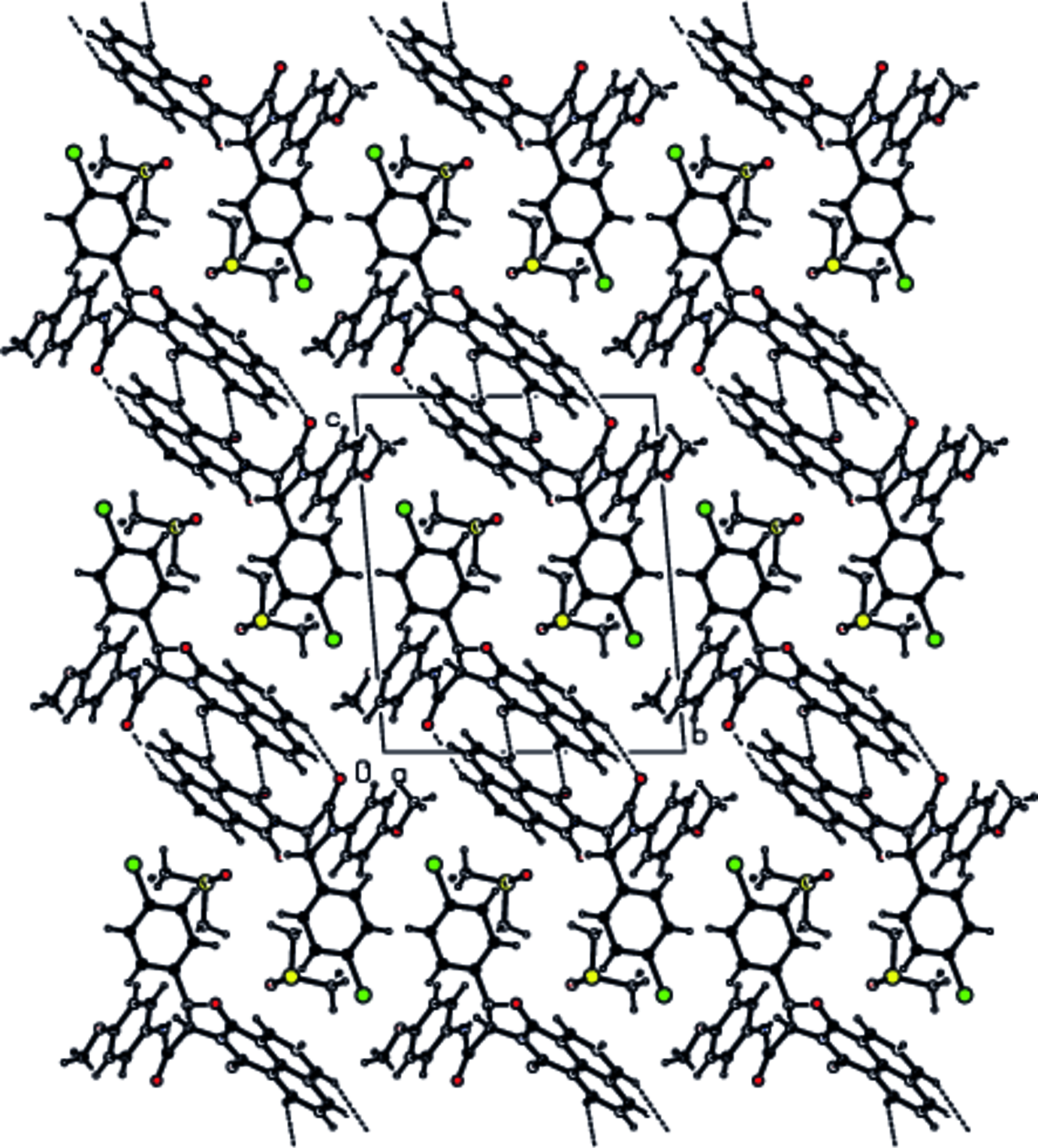

Supplement: Supplementary file 5 [file e-71-0o129-fig2.tif]

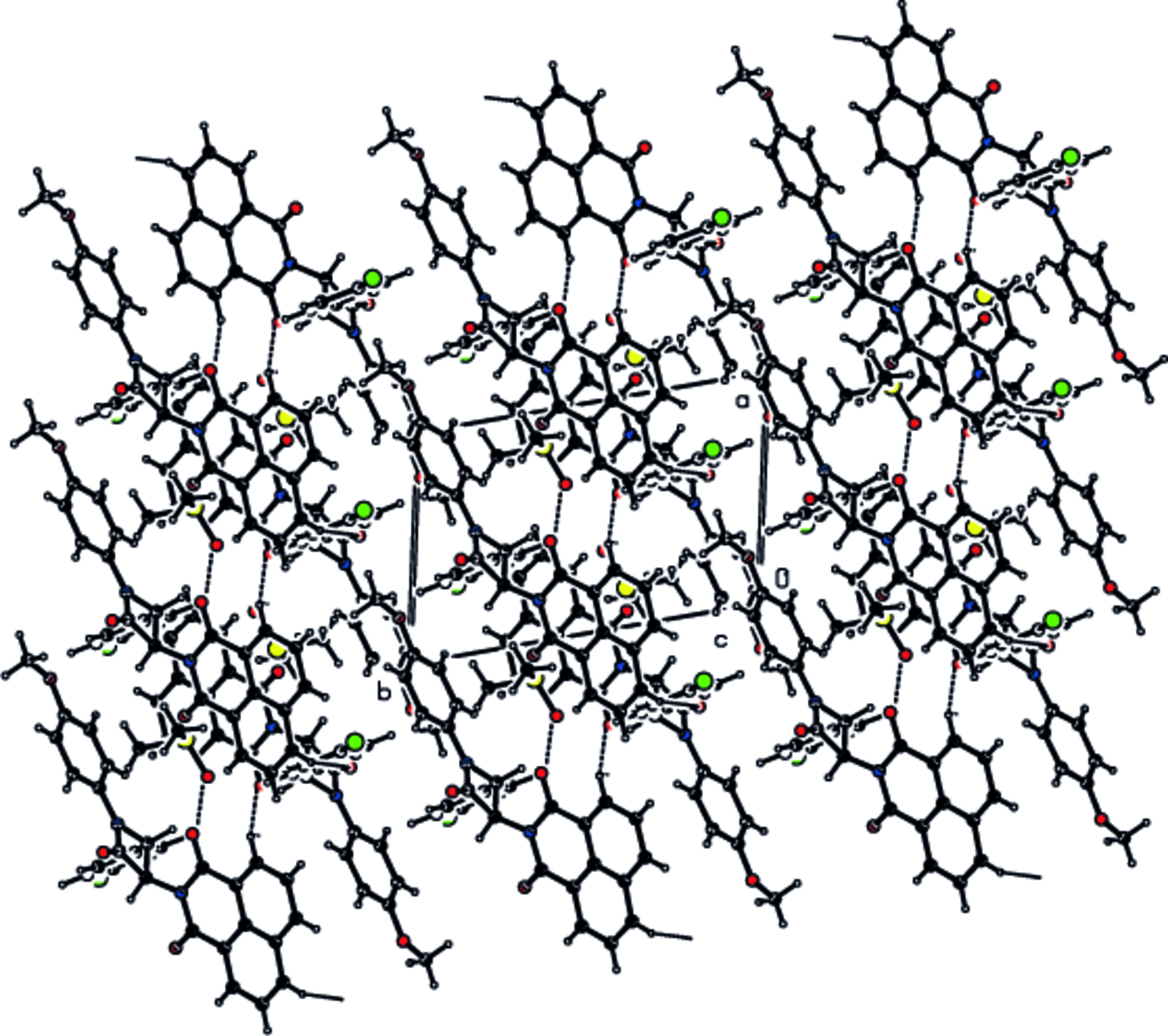

Supplement: Supplementary file 6 [file e-71-0o129-fig3.tif]
